# The Role of MicroRNA-206 in the Regulation of Diabetic Wound Healing via Hypoxia-Inducible Factor 1-Alpha

**DOI:** 10.1007/s10528-024-10759-9

**Published:** 2024-03-06

**Authors:** Zeming Bai, Dapeng Zhou, Kai Tao, Feng Lin, Hongyi Wang, Haiwei Sun, Ruidi Liu, Zhe Li

**Affiliations:** Burn and Plastic Surgery Department, General Hospital of Northern Theater Command, Shenyang, 110000 China

**Keywords:** Diabetic wounds, Hyperglycemia, HIF-1α, miR-206, VEGF

## Abstract

Successful wound healing in diabetic patients is hindered by dysregulated miRNA expression. This study aimed to investigate the abnormal expression of miRNAs in diabetic wound healing and the potential therapeutic role of modulating the miR-206/HIF-1α pathway. MicroRNA assays were used to identify differentially expressed miRNAs in diabetic wound sites and adjacent areas. In vitro models and a rat diabetic model were established to evaluate the effects of miR-206 on HIF-1α regulation and wound healing. The study revealed differential expression of miR-206 in diabetic wound tissues, its interaction with HIF-1α, and the inhibitory effect of miR-206 on cell growth under high glucose conditions. Modulating the miR-206/HIF-1α pathway using miR-206 antagomir promoted HIF-1α, CD34, and VEGF expression, ultimately enhancing diabetic wound healing.

## Introduction

With the change of demographic characteristics, the incidence rate of diabetes is increasing rapidly with a 9.3% global prevalence recorded in 2019 that is estimated to reach 10.3% in 2030 (Saeedi et al. 2019). About 15–20% diabetic patients are at risk of lifetime suffering from chronic non-healing wound, such as diabetic foot ulcers (DFU), of which costing Medicare around 9–13 billion USD in the United States and costing National Health Service around 1 billion GBP in the United Kingdom (Geraghty and LaPorta 2019; Jodheea-Jutton et al. 2022; Lu et al. 2020). As a common refractory complication, diabetic wound healing is a result of complicated pathophysiology, which involves local skin ulceration, infiltration and inflammatory reactions, granulation, cell proliferation, wound edge contraction, and alteration of tissue shape (Falanga 2004; Spampinato et al. 2020). Diabetic wounds are difficult to treat, and greatly affect the routine life due to the induced local pain and dysfunction (Cerny et al. 2021). To date, no rapid and effective treatment is available for complete cure of diabetic wound (Burgess et al., 2021), and thus necessitates the development of new therapeutic treatment approaches.

Recently, the role of microRNAs (miRNAs), the endogenous and non-coding single-stranded 18–23 nucleotides long RNA molecules (Nie et al. 2020), has been revealed in wound healing. These miRNAs play a vital role in re-epithelization, angiogenesis, collagen deposition, and other important links in wound repair (Chen et al. 2021). Many research groups are focusing to explore the role of miRNAs in wound healing. A study showed that, the upregulation of miR-129 or -335 favors diabetic wound healing (Wang et al. 2018). In another study, the knockout of miR-20b-5p in diabetes-induced mice promoted wound healing and angiogenesis. In vivo studies in diabetic patients showed that miR-20b-5p was highly enriched in exudates and could be transferred to vascular endothelial cells (Xiong et al. 2020). It has also been shown that miR-210 regulates cell-cycle and mitochondrial respiratory proteins in diabetic tissues through anti-wound repair mechanisms (Dallas et al. 2019).

Hypoxia inducible factor (HIF-1) is a key regulator of oxygen homeostasis and an important determinant of healing outcomes. It participates in pathophysiological processes such as energy metabolism, angiogenesis, proliferation, migration, and apoptosis (Yang et al. 2017). In nucleus, the hypoxia-inducible factor 1-alpha (i.e., HIF-1α) of the heterodimeric transcription factor hypoxia-inducible factor 1 (HIF-1) regulates the vascular endothelial growth factor (VEGF), erythropoietin, platelet-derived growth factor, fibroblast growth factor, and transforming growth factor β. Specifically, HIF-1α directly binds to VEGF, the strongest stimulator for promoting angiogenesis, and increases its expression. It activates the signaling pathway through receptor binding of endothelial cell membrane and further induces the expression of angiogenic genes, which in turn contribute to repair the damaged blood vessels and produce new vessels at the wound site (de Mattos et al. 2021; Zhu et al. 2019). It has also been reported that miR-217 downregulated the HIF-1α/VEGF pathway, and promoted angiogenesis and decreased inflammation in DFU rats, which ultimately led to improved healing of diabetic ulcer area (Lin et al. 2019). In addition, it has been shown that miR-15b and miR-21a have an effect on modulating the expression of HIF-1α in wound tissues during the pathophysiological stage of granulation and inflammation and further accelerates the secretion of VEGF-A (Cakmak et al. 2018).

This study was aimed to assess the abnormal expression of miRNAs in wound tissues of diabetic patients, and found high expression of miR-206 in diabetic wound tissues. α We further revealed the mechanism underlying the miR-206 regulate diabetic wound healing.

## Materials and Methods

### Eligibility Criteria and Sample Collection

The study included a total of six patients, and their inclusion criteria were: 50–69 years old, history of chronic foot ulcers with non-healing trauma, under insulin control, fasting glucose level > 7 mmol/L, two-hour postprandial glucose level > 11.1 mmol/L, HbA1c level > 7 mg/dl. Furthermore, the included patients had impaired fasting glucose (IFG), impaired glucose tolerance (IGT), met the diagnostic criteria for DFU, ankle-brachial index values between 0.9 and 1.3, and foot trauma lasted approximately 2–6 weeks. It was also ensured that the patients neither received any hormone, drug, radiotherapy, or other treatment before operation nor have any systemic disease, and fully cooperate during the study period. On the other hand, patients with ulcers due to trauma lower limb vascular disease, ulcerated malignancy, and untreated osteomyelitis or cellulitis were excluded from the study. For analysis, diabetic ulcer tissues and normal skin tissue samples (i.e., control) were collected from all patients.

### Ethical Approval

The study was approved by the Medical Ethics Committee of the General Hospital of Northern Theater Command (Shenyang, China; R20210209). An informed consent document was signed by all patients before sample collection.

### MicroRNA Array Analysis

The diabetic ulcer tissue and adjacent normal skin tissue samples were homogenized in 1 mL Trizol and incubated for 3 min at room temperature. Total RNA (including short RNA) (< 200 bp) was extracted from all samples by using the RNA micro elution Kit (Qiagen, Wenluo, the Netherlands). MiRNA arrays 3.0 software (Affymetrix, Santa Clara, CA, USA) was used for analysis of the extracted RNA samples.

### Animal Model

The animal protocol of this study was reviewed and approved by the Institutional Animal Care and Use Committee (IACUC) according to the guidelines of the Institutional Ethics Committee at Northern Theater General Hospital (Shenyang, China; R20210316). The animal study included a total of 6-8-week-old 36 healthy Sprague Dawley (SD) rats (weight 180–240 g; 1:1 male and female), provided by the Animal Laboratory of the General Hospital of the Northern Theater (purchased from Liaoning Changsheng Biotechnology Co., Ltd., Animal License No.: SCXK (Liao) 2015-0001). The animals were housed in UV-sterilized room with light/dark cycle of 12 h at 21 ± 1 °C and indoor humidity of 50 ± 10%. The animals had free access to feed. For study, the 36 SD rates were divided into two main groups: including 6 in the control group and 30 for development of diabetes models. The rats underwent a 12 h water fasting before modelling and the blood glucose was measured by blood glucose meter before injection. Basal blood glucose < 8.9 mmol/L before induction was considered to be at the glycemic standard. After the blood glucose met the standard, the rats were given a single intraperitoneal injection of streptozotocin (STZ) according to the weight of the rats at a dose of 60 mg/kg. After 72 h of STZ injection, the blood was collected by tail cutting method for determination of random blood glucose level. The rats with blood glucose ≥ 16.7 mmol/L were considered as the successfully developed models. Among the 30 diabetic rats, 12 were injected with 50 µM scramble (control for injected miRNA; Shanghai Hengfei Biotechnology Co. Ltd., Shanghai, China), 12 were injected 50 µM miR-206 antagomir (Shanghai Hengfei Biotechnology Co., Ltd., Shanghai, China), and 6 were injected with 50 µM saline at 3 random points around the wound edge after injury. The wound area of 3 randomly chosen rats in scramble group and the miR-206 antagomir group was measured by digital camera at day 3, 7, and 14 after treatment. After 4 weeks, all rats were anesthetized and injected intraperitoneally with 40 mg/kg pentobarbital sodium, and the hairs in the central area of the back were shaved with an electric shaver. The central area of the spine was marked as the square area with 1.5 cm length. The whole skin of diabetic rats was removed by marking line. The rats were euthanized if they had 15–20% weight loss, have cachexia, complete loss of appetite for 5 consecutive days or have poor appetite for 7 days, unable to eat and drink, or cannot stand or reluctant to stand for 24 h, and showed adverse reactions, such as organ infection. For RNA extraction or immunocytochemical analysis, the rats were anesthetized with 100 mg/kg of pentobarbital sodium and culled by cervical dislocation at the corresponding time point, and the back wound of each rat and 2 mm adjacent normal tissue was immediately dissected.

### Cell Culture and Treatment

Human foreskin fibroblasts 1 (HFF-1; Mingzhou Biotechnology Co., Ltd., Ningbo, China) and vascular endothelial cell (VE; Mingzhou Biotechnology Co., Ltd., Ningbo, China) were cultured in Dulbecco’s Modified Eagle medium (DMEM) containing 10% fetal bovine serum (FBS). The culture flasks were incubated at 37 °C in humidified incubator (5% CO_2_; 95% air) and the growth and adhesion of cells were observed daily. To establish a glucose model in vitro, the cells were divided into three groups according to treatment with different glucose concentrations (0, 20, and 40 mmol/L).

### Cell Transfection

To explore the relative relationship between miR-206 and HIF-1α, the in vitro experimental groups were divided into different groups, including: (1) miRNA scramble, named as scramble group; (2) miR-206 group; (3) miRNA inhibitor group; (4) miRNA scramble + lentivirus vector, named as scramble + vector group; (5) miRNA scramble + lentivirus HIF-1α, labeled as scramble + HIF-1α group; (6) miR-206 + lentivirus vector, labeled as miR-206 + vector group; and (7) miR-206 + lentivirus HIF-1α, labeled as miR-206 + HIF-1α group. After the cells reached 90% confluence in the 6-well plate, the cells are transfected according to the experimental design. The lipo2000 (Thermo Fisher Scientific, China) was also added to the cells. After 20 min of incubation at room temperature, the cells were washed and further incubated with serum-free medium for 5 h. After wards, the cells were incubated with serum-containing medium in a CO_2_ incubator at 37 °C for 48 h to observe the degree of transfection.

### Quantitative Real-Time Polymerase Chain Reaction

Total RNAs were extracted from in vivo tissues or cell lysates following the manufacturers’ instructions. Briefly, the tissues or cell lysates were homogenized in 1 mL Trizol (YITA, Biotechnology Co., Ltd., Beijing, China) and incubated for 3 min at room temperature. The samples were then mixed with anhydrous ethanol, and centrifuged at 10,000 rpm for 10 min. After centrifugation, the supernatant was resuspended in RWA buffer (YITA Biotechnology Co., Ltd., Beijing, China), centrifuged, and dried. The obtained RNA was then eluted with DEPC water.

To reverse transcribe RNA into cDNA, the real time reaction kit (Promega, Beijing, China) was used according to the manufactures’ instructions. After that, Mx3000p real-time PCR system (Applied Biosystems, Beijing, China) was used for real-time quantitative PCR (qPCR). The primers (Shanghai Kanglang Biotechnology Co., Ltd., Shanghai, China) used in this study are listed in Table 1. The expression level of target genes was calculated by the 2^−∆∆CT^ method (Al-Ouqaili et al. 2020).

**Table 1:** The names and sequences of forward and reverse primers used for qRT-PCR

Primer	Forward	Reverse
HIF-1α	5′-TATGAGCCAGAAGAACTTTTAGGC-3′	5′- CACCTCTTTTGGCAAGCATCCTG − 3′
GADPH	5′-AGCCACATCGCTCAGACAC-3′	5′-GCCCAATACGACCAA ATCC-3′
miR-206	5′ GGAATGTAAGGAAGTGTGTG-3′	5′-GAACATGTCTGCGTATCTC-3′
U6	5′ -CGCTTCGGCAGCACATATAC-3′	5′-AAATATGGAACGCTTCACGA-3′

### Western Blot

Rat tissue samples and cell samples were lysed with RIPA lysate buffer (AITA Biotechnology Co., LTD., Beijing, China). Briefly, 50 µg protein samples were resolved *via* 10% sodium dodecyl sulfate-polyacrylamide gel electrophoresis (SDS-PAGE) and transferred to nitrocellulose membrane (Sigma–Aldrich, Saint Louis, MO, USA) by electro imprinting. Membranes were blocked in 5% skimmed milk and incubated overnight at 4 °C with primary antibodies, including anti-HIF-1α (1:1000, Santa Cruz biotechnology, sc-13,515) and anti-GAPDH (1:1000, Santa Cruz biotechnology, sc-32,233). The membranes were then incubated with secondary antibody (1:5000) at room temperature for 1 h. The protein bands were visualized with western lighting ultra instrument (ECL, Pierce Technology, Shanghai, China). The relative expression of proteins were normalized to GADPH by using the ImageJ software (NIH Image, United States).

### MTT Assay

HFF-1 and VE cells were seeded on 96-well plate at a density of 1 × 10^4^ cells/well, and either treated with different concentrations of glucose or transfected with different vectors according to the experimental design. After 0, 12, 24, 36, and 48 h, 10 µL of 5 mg/mL MTT solution was added and the medium was replaced with 150 µL of dimethyl sulfoxide solution after incubation for 4 h at 37 °C. Microplate reader (Bio-Rad Laboratories, Shanghai, China) (Yang et al. 2010) was used measure the optical density at 490 nm to determine cell proliferation.

### Immunofluorescence Staining

The HFF-1 and VE cells at a density of 2 × 10^5^ cells/well were seeded in 96-well plates and divided into different treatment groups. Cells were fixed with 4% paraformaldehyde for 15 min, and washed three times with phosphate buffer saline (PBS). The cells were incubated with 5% Triton X-100 for 20 min and then 10% goat serum at room temperature for 30 min. Afterwards, the primary antibody mouse anti-HIF-1αwas incubated overnight at 4 °C. The cells were washed with PBS and incubated with secondary antibody goat anti-mouse IgG-HRP. The cells were then incubated in DAPI for 5 min. Finally, the cells were visualized and imaged under confocal laser scanning microscope (Leica, Germany) at 400×.

### Immunohistochemical Staining

After fixing in 10% formaldehyde on pathological slides, the tissue samples were deparaffinized in xylene, rehydrated in decreasing gradient ethanol concentrations, and immunohistochemically stained, as reported previously (Patel and Chaudhary 2012). The slides were blocked with 0.5% BSA for 30 min, and incubated overnight at 4 °C with primary antibodies, including mouse anti-CD34, mouse anti-HIF-1α, or rabbit anti-VEGF. After washing with PBS, the slides were incubated with secondary antibodies (Vector Laboratories, Burlingame, CA, USA) for 1 h at room temperature. The slides were then treated with avidin-biotin complex (Vector Laboratories, Burlingame, CA, USA) for 30 min at room temperature. The slides were counterstained with Haematoxylin and images were taken by Nikon Eclipse E600 microscope (Nikon Instruments, Tokyo, Japan). The positive expression is presented as brownish yellow color. With the brownish yellow positive expression signal as the marker, five areas on each slide were randomly selected at 200x and the average optical density of the slides from two groups culled at day 3, 7, and 14 was analyzed by IPP software, according to below formula.

$$\text{M}\text{e}\text{a}\text{n}\,\text{d}\text{e}\text{n}\text{s}\text{i}\text{t}\text{y}\,=\,\frac{\text{O}\text{p}\text{t}\text{i}\text{c}\text{a}\text{l}\,\text{d}\text{e}\text{n}\text{s}\text{i}\text{t}\text{y}}{\text{M}\text{e}\text{a}\text{s}\text{u}\text{r}\text{m}\text{e}\text{n}\text{t}\,\text{a}\text{r}\text{e}\text{a}}$$

### Luciferase Report Analysis

The miR-206 complementary site in the 3’-UTR of HIF-1α wild-type (WT) or its mutant (MUT) was PCR amplified and cloned into the pEZX vector. Afterwards, the pEZX-HIF-1α -3’-UTR-WT or pEZX-HIF-1α -3’-UTR-MUT was co-transfected with 10 nM miR-206 or its negative control (scramble) in 96-well plate for 24 or 48 h. After 24 or 48 h of transfection, the luciferase activity was determined by using the dual luciferase reporter assay system performed according to the manufacturer’s instructions (Promega Corporation, Madison, WI, USA).

### Statistical Analysis

All in vitro experiments were performed in triplicate and repeated at least three times. GraphPad Prism 7 (Graphpad software, CA) was used to analyze the data and all data were represented as the mean ± standard error of the mean (SEM). Statistical analysis was performed with Pearson correlation coefficient, Student’s *t*-tests, or one/two-way analysis of variance (ANOVA) with Bonferroni’s multiple comparison post-hoc test. *P* < 0.05 was considered statistically significant.

## Results

### miR-206 was Upregulated in Human Diabetic Wound Tissues

**Fig. 1:**
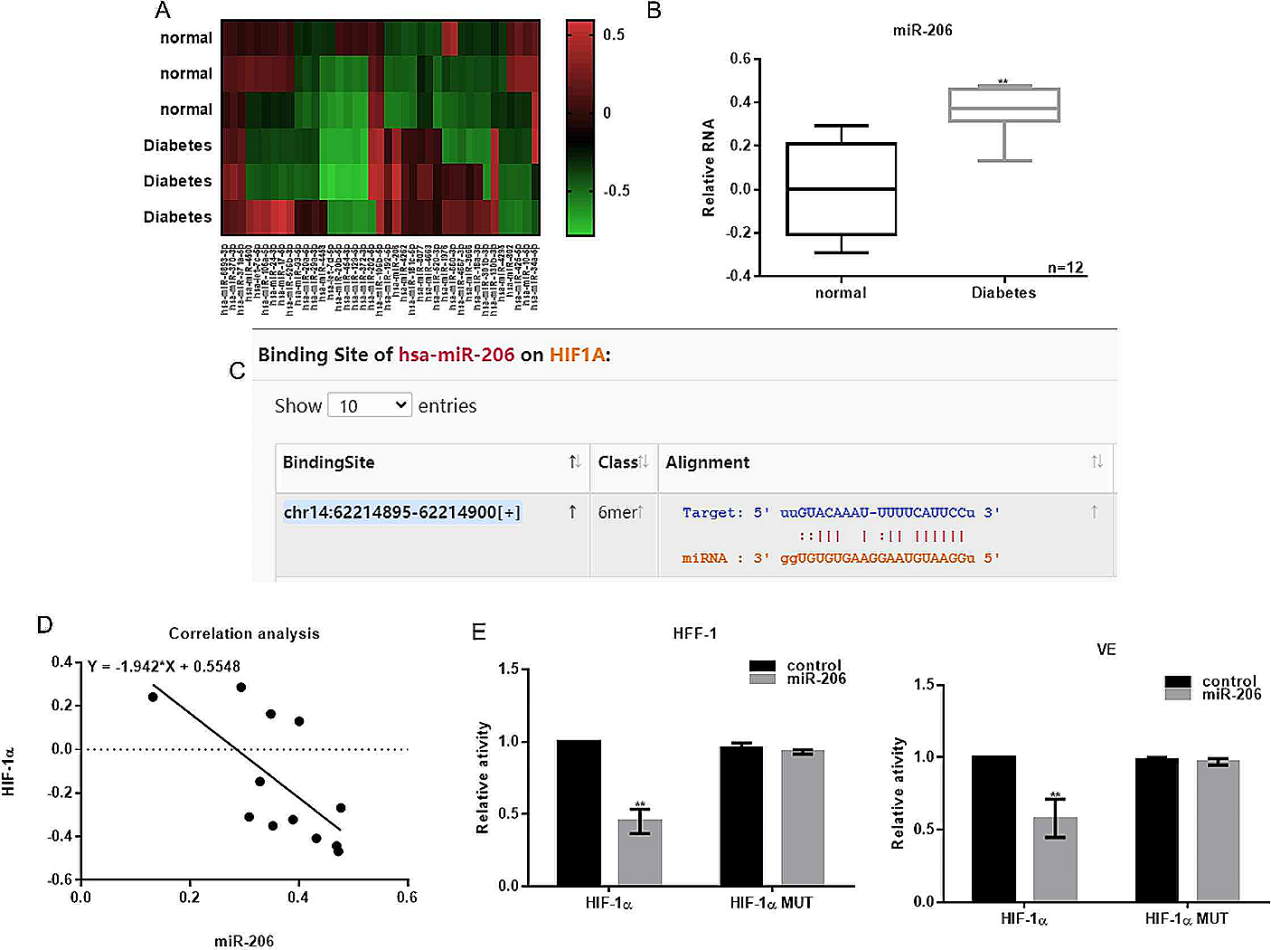
MiR-206 was upregulated in diabetic wound tissue in vivo. (**A**) Differential expression of miRNAs in diabetic tissues and adjacent normal tissues was assessed by miRNA microarray analysis. (**B**) Expression of miR-206 of diabetic tissues and 6 adjacent normal tissues was measured. **p < 0.05. (**C**) Binding sites between miR-206 and HIF-1α. (**D**) Correlation between miR-206 and HIF-1α was estimated. Relative dual-luciferase activity of HIF-1α in (**E**) HFF-1 and (**F**) VE cells expressing miR-206. **p < 0.05, vs. scramble

The microRNA array was carried out to investigate the expression of miRNAs in wound tissues of diabetic and normal tissues. Figure 1A shows that a variety of miRNAs, including miR-20b-5p, miR-454-3p, miR-129-5p, miR-372-3p, miR-192-5p, miR-206, miR-4262, miR-181c-5p, miR-8077, miR-4663, miR-520-3p, miR-130b-3p, miR-802, miR-425-5p, and miR-10-5p, were differentially expressed. Among these, the expression of miR-206 was higher in diabetic tissues than the adjacent normal tissues (Fig. 1B). Furthermore, the prediction from Targetscan database showed that miR-206 was very likely to bind with the 3’-UTR of HIF-1α (Fig. 1C). Correlation analysis showed a negative correlation between miR-206 and HIF-1α (Fig. 1D). The results of luciferase reporter gene experiment in HFF-1 and VE cell lines showed that miR-206 downregulated the activity of HIF-1α. However, the inhibition effect of miR-206 was disappeared after mutating their binding sites (Fig. 1E). These finding suggest that miRNAs, especially miR-206, were overexpressed in the diabetic tissues, and HIF-1α is downregulated when miR-206 is overexpressed.

### High Glucose Influenced Cell Viability and Expression of miR-206 and HIF-1α

**Fig. 2:**
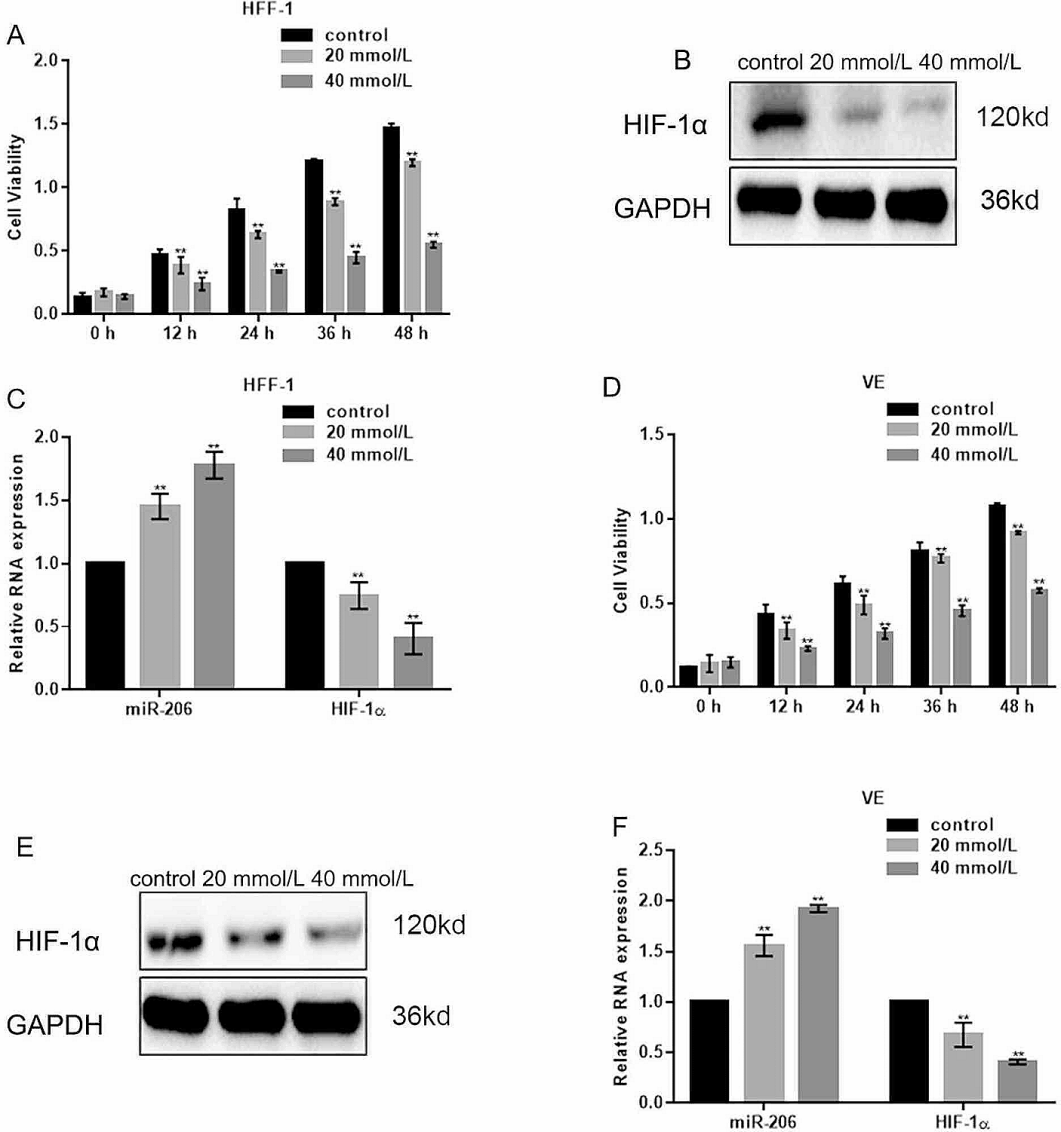
High glucose concentration affected cell viability and expression of miR-206 and HIF-1α. (**A**) Viability of HFF-1 cells after 24, 36, and 48 h of culture was significantly decreased with the increased concentration of glucose. **p < 0.05, vs. control. (**B**) Expression of HIF-1α in HFF-1 cells was decreased at high glucose concentration. (**C**) Expression of miR-206 in 40 mmol/L glucose was significantly increased while the mRNA level of HIF-1α was significantly decreased compared to the control group. **p < 0.05, vs. control. (**D**) Viability of VE cells was significantly decreased after treatment with 0, 20, and 40 mmol/L glucose. **p < 0.05, vs. control. (**E**) Increasing glucose concentration decreased the expression of HIF-1α in VE cells. (**F**) mRNA expression of miR-206 under high glucose condition was significantly increased while the mRNA level of HIF-1α was significantly decreased compared to the control group in VE cells. **p < 0.05, vs. control

The effect of high glucose concentration on the viability of fibroblasts and vascular endothelial cells was assessed by MTT assay. The results showed that the viability of HFF-1 cells (Fig. 2A) and VE cells (Fig. 2D) was significantly decreased in the presence of high glucose concentration at different time points, including 12 h, 24 h, 36 h, and 48 h. Compared to the control group (no glucose), the proliferative activity in high glucose group (40 mmol/L) was decreased nearly to half starting from 24 h (Fig. 2A, D). These results are in accordance with the western blot which showed that the expression of HIF-1α was decreased with the increase of glucose concentration in both HFF-1 cells (Fig. 2B) and VE cells (Fig. 2E). The qPCR results showed that in both HFF-1 cells (Fig. 2C) and VE (Fig. 2F), the relative miRNA expression of miR-206 in 40 mmol/L was significantly increased as compared to the lower concentration of glucose group and the control group, while the miRNA level of HIF-1α was significantly decreased by about 50% compared to the control group. These findings indicate that high glucose concentration, which correlates the diabetes, has a significant negative impact on the cell viability, accompanied by upregulation of miR-206 and downregulation of HIF-1α.

### miR-206 Inhibited Cell Proliferation

**Fig. 3:**
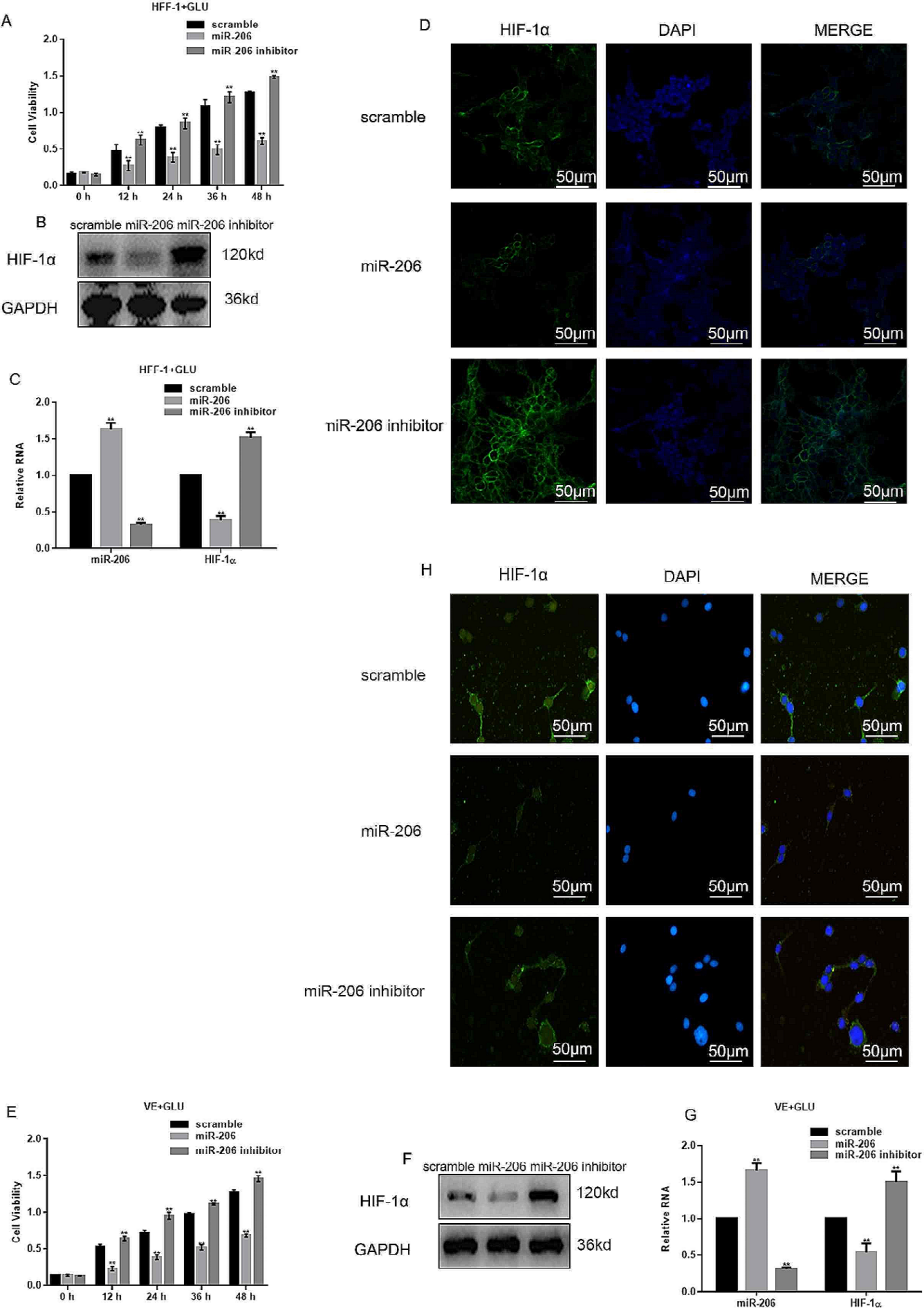
MiR-206 inhibited the proliferation of cells. (**A**) Viability of HFF-1 cells was significantly increased with the inhibition of miR-206. **p < 0.05, vs. scramble. (**B-D**) HIF-1α was detected by immunofluorescence staining, western blot, and RT-PCR showing that HIF-1α expression was up-regulated with the miR-206 suppression. **p < 0.05, vs. scramble. Original magnification ×200 (**E**) MTT assay showed high viability of VE cells when miR-206 was inhibited. **p < 0.05, vs. scramble. (**F, G**) Western blot and qRT-PCR reveal that inhibition of miR-206 significantly improved the expression of HIF-1α. **p < 0.05, vs. scramble. (**H**) Expression of HIF-1α in miR-206 or miR-206 inhibitor transduced cells was assessed by immunocytochemistry, showing that the expression of HIF-1α was decreased in the presence of miR-206 and increased in the presence of miR-206 inhibitor

To further explore the effect of miR-206 and its impact on HIF-1α, we either overexpressed or inhibited miR-206 in HFF-1 cells and VE cells. With the inhibition of miR-206, the cell viability in high glucose group was significantly increased when compared to the cells overexpressed with miR-206 (Fig. 3A, E). These results suggests that miR206 plays an important role on cell proliferation in diabetics. Western blot and qPCR results also revealed that in both HFF-1 cells (Fig. 3B, C) and VE cells (Fig. 3F, G), the expression of HIF-1α was significantly increased when miR-206 was inhibited. The immunofluorescence staining of HFF-1 cells showed that miR-206 decreased the expression of HIF-1α, and when HFF-1 cells were treated with miR-206 inhibitor, these demonstrated an increase expression of HIF-1α (Fig. 3D). Furthermore, when VE cells were overexpressed with miR-206, the intensity of HIF-1α was decreased while when introduced with miR-206 inhibitor, there was a trend that the expression of HIF-1α was reversed (Fig. 3H). Furthermore, DAPI staining demonstrated that the number of cells were decreased when miR-206 was over-produced (Fig. 3D, H) which was in accordance with the results of MTT assay (Fig. 3A, E). Overall, these results suggest that miR-206 inhibited cell proliferation and decreased the expression of HIF-1α.

### miR-206 Inhibited Cell Proliferation by Suppressing HIF-1α

**Fig. 4:**
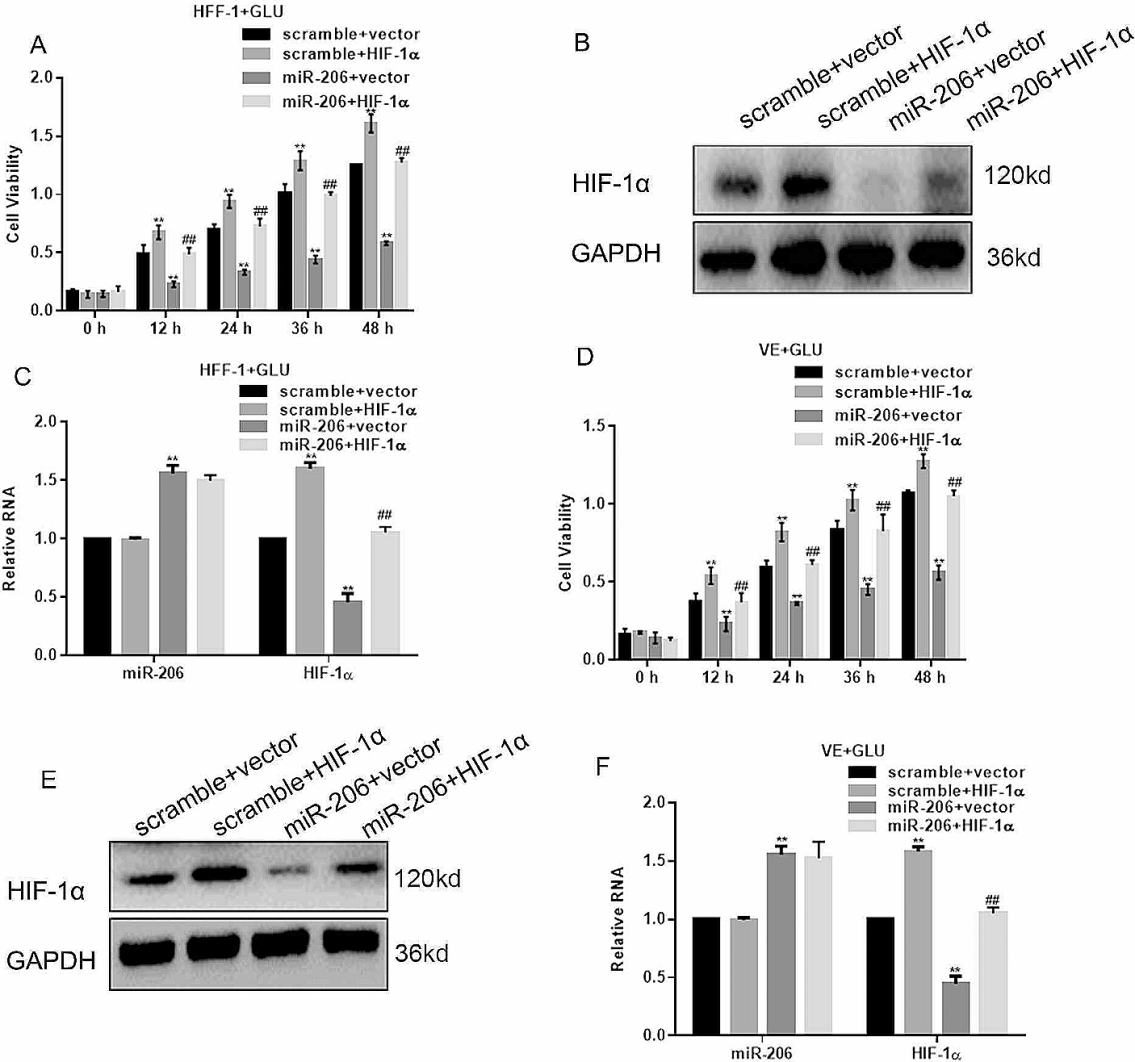
MiR-206 inhibited cells proliferation by inhibiting HIF-1α. (**A**) HFF-1 cells showed a decreased inhibitory effect of miR-206 when co-transfected with HIF-1α.**p < 0.05, vs. scramble + vector, ##*p* < 0.05, vs. miR-206 + vector. (**B, C**) Overexpression of miR-206 decreased the expression of HIF-1α but the expression was improved when co-transfected with HIF-1α in HFF-1 cells. **p < 0.05, vs. scramble + vector, ##*p* < 0.05, vs. miR-206 + vector. (**D**) MTT assay showed the viability of the indicated VE cells. **p < 0.05, vs. scramble + vector, ##*p* < 0.05, vs. miR-206 + vector. (**E, F**) Overexpression of miR-206 decreased the expression of HIF-1α but the effect was reversed when co-transfected with HIF-1α in VE cells. **p < 0.05, vs. scramble + vector, ##*p* < 0.05, vs. miR-206 + vector

We also assessed the impact of miR-206 and HIF-1α co-transfection in high glucose medium. MTT results showed that HIF-1α promoted the proliferation of HFF-1 cells starting at 12 h compared to the control group (Fig. 4A). Additionally, the inhibitory effect of miR-206 was significantly weakened when simultaneously introduced with HIF-1α, with an approximate 3-fold increase after 48 h compared with the miR-206 overexpression only (Fig. 4A). Moreover, the data from Western blot and qPCR showed that miR-206 overexpression downregulated the expression of HIF-1α but with the co-transfection of HIF-1α, the expression was restored (Fig. 4B, C). Similar results were shown by the VE cells (Fig. 4D-F). These findings suggest that overexpression of HIF-1α rescued the inhibitory effect of miR-206 on cell proliferation, and that miR-206 affected cell proliferation by regulating the expression of HIF-1α.

### miR-206 Antagomir Promoted Wound Healing in Diabetic Rats

**Fig. 5:**
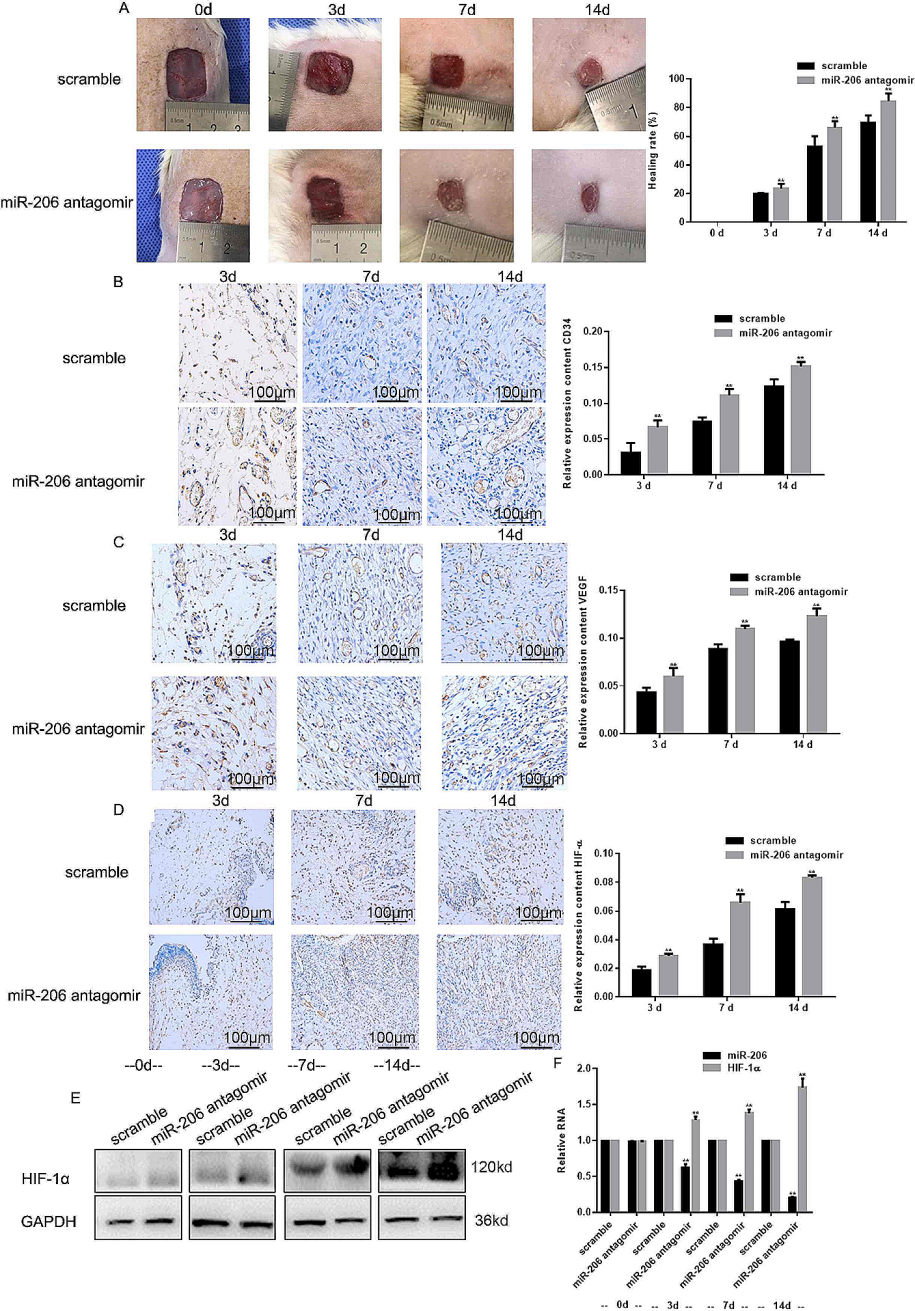
MiR-206 antagomir promoted wound healing in diabetic rats. (**A**) Wound healing rate of miR-206 antagomir group was significantly increased compared to the scramble group at day 3, 7, and 14. (**B, C**) Relative mRNA expression of CD34 and VEGF of miR-206 antagomir group and scramble group was significantly increased at day 3, 7, and 14. Original magnification ×200 (**D-F**) Relative mRNA expression of HIF-1α was up-regulated in tissues of miR-206 antagomir group. **p < 0.05. vs. scramble group. Original magnification ×200

From the in vivo study, the wound healing rate of the group administered with miR-206 antagomir was significantly enhanced compared to the scramble group at 14 days post-surgery (Fig. 5A). In addition, the relative expression of CD34 and VEGF in miR-206 antagomir group was significantly increased compared to the scramble group at day 3, 7, and 14 (Fig. 5B, C). Furthermore, when administered with miR-206 antagomir in vivo, the expression of HIF-1α in vivo was also up-regulated (Fig. 5D-F). In summary, these results suggest that miR-206 might affect wound healing in diabetic rats *via* the HIF-1α/VEGF pathway.

## Discussion

Diabetes is a metabolic disease, where 19–35% of diabetic wounds do not heal and lead to increase the risk of amputation (Chen et al. 2021a, b; Deng et al. 2021). This necessitates the exploration and development of effective and reliable therapeutic treatment approaches. In this study, we determined the microRNA profiles in diabetic tissue and screened out the target miR-206 with the prediction of underlying HIF-1α pathway. We found that miR-206 antagomir promoted the expression of HIF-1α and VEGF, and promoted wound healing in diabetic rats in vivo. Our findings suggest that miR-206 could affect angiogenesis and proliferation by regulating the expression of HIF-1α, and further regulate wound healing.

To date, several studies have reported various roles of miR-206, such as regulating the growth and migration of hepatocellular carcinoma cells by affecting Sox9 (Lin et al. 2021), participating in the injury of endothelial cells (Gao et al. 2021; Al-Kanaan et al. 2020), and certain early diagnostic significance for skeletal muscle injury (Yamaura et al. 2020). Vinod and colleagues showed resistance to impaired glucose metabolism in response to high-fat diet (HFD) in mice that effectively knockout the expression of miR-206. These findings suggest the inhibitory effect of miR-206 on diabetes. They further explored the correlating glucokinase (GK) pathway, and showed that the knockout of miR-206 increased the activity of GK in islets and liver (Vinod et al. 2016). In another study, Delfan et al. found that miR-206 activated apoptosis pathways through downregulation of HSP60, an anti-apoptosis protein, in vitro (Delfan et al. 2022). Interestingly, their group also established a glucose model which treated C2C12 cells with different concentrations of glucose. They measured the cell viability and concluded that high glucose induced miR-206 expression and reduced cell viability, which is in accordance to the findings of this study where high glucose concentration induced the expression of miR-2016 and reduced the viability of HFF-1 and VE cells.

Although there are some studies focusing the role of miR-206 on diabetics, some other studies have explored the underlying potential mechanism targeting the HIF-1α/VEGF. Lin et al. reported an abnormal expression of miR-217 which was found in the tissues of DFU (Lin et al. 2019; Al-Ouqaili et al. 2017). They established DFU rat model and treated that with miR-217 inhibitor and/or HIF-1α siRNA. The found that DFU rats treated with miR-217 inhibitor significantly reduced the expression of HIF-1α and VEGF, indicating that miR-217 could regulate the angiogenesis of diabetic rats by modulating the HIF-1α/VEGF pathway. Studies have also showed a correlation between miR-206 and HIF-1α in other diseases, such as colorectal cancer, lung cancer, and breast cancer (Pan et al. 2017; Xu et al. 2018; Xue et al. 2016; Yu et al. 2021). However, very few studies have been carried out on the role of miR-206 and its effect on HIF-1α in diabetic models. Moreover, the key mechanism underlying HIF-1α/VEGF pathway is crucial that VEGF is also a downstream target of HIF-1α. In in vivo studies, the presence of skin wounds elevated the expression of HIF-1α which further increased the angiogenic factor, such as VEGF (Chen et al. 2012; Yang et al. 2019). The upregulation of VEGF triggers the migration and proliferation of VE cells which play an important role in angiogenesis and ultimately enhances the local blood supply to skin wounds. Therefore, our study provides a novel sight of modulating miR-206 *via* HIF-1α/VEGF pathways to potentially affect angiogenesis in the field of diabetic wound healing.

Currently, several miRNA drugs are under human clinical trials, which have shown promising outcomes (Reid et al. 2016; Rupaimoole and Slack 2017). For example, Beg et al. found in Phase I study encapsulated MRX34, a liposomal nanoparticle formulation, with the miR-34a mimic and evaluated for its activity in 75 patients with advanced malignancies (Beg et al. 2017). The toxicity analysis did not show any adverse effects and two participants demonstrated partial responses. Therefore, miRNA treatment could be used as an alternative therapy and there is a strong possibility that our proposed strategy of treating diabetic wounds by modulating the expression of miR-206 is prospective to be translated into clinic in the future.

A potential limitation of this study is that we only demonstrated the representative images of western blot and immunohistochemistry here. In addition, in terms of in vivo study, behavior tests, which relates to the wound healing, could be carried out. For example, pain which is a physical and psychological stressor, has an impact on wound healing (Gouin and Kiecolt-Glaser 2011). In this case, von-frey can be used to assess the pain in animal model in order to measure the effect on wound healing in vivo. Another limitation of this study could be the small sample size of only six human participants. Therefore, more experiments could be designed and carried out to confirm our hypothesis that modulating miR-206 expression has a potential effect on diabetic induced wound healing in vivo on large human sample size.

In conclusion, miR-206 was differentially expressed in diabetic wounds and the adjacent normal tissues, suggesting its important role in wound healing. The expression of miR-206 was significantly upregulated in wound tissues of diabetic rats, and it inhibited the fibroblast growth and suppressed wound angiogenesis by regulating the expression of HIF-1α. And the downregulation of miR-206 promoted wound healing in fibroblasts and vascular endothelial cell culture in vitro and in the diabetic rat model in vivo by promoting the expression of VEGF and HIF-1α. Therefore, miR-206 could be a potential therapeutic target and modulation of its expression could be a potential therapeutic approach for treating diabetic wounds.

## Data Availability

All data from this article can be supplied by the author upon reasonable request. The data associated with this manuscript have been submitted to an online: GSE 188783.

## References

[CR39] Al-Kanaan BM, Al-Ouqaili MTS, Al-Rawi KFA (2020) Comparative study of the molecular, biochemical, and other parameters in Iraqi hepatitis B patients. Drug Invent Today 14(6):870–881

[CR40] Al-Ouqaili MTS et al (2017) Depending On HPLC and PCR, detection of aflatoxigenic and non-aflatoxigenic strains of *Aspergillus flavus* isolated from some clinical and environmental sources. Asian J Pharm 12(1):40–48. 10.13140/RG.2.2.30869.73442

[CR41] Al-Ouqaili MTS, Majeed YH, Al-Ani SK (2020) SEN virus genotype H distribution in β-thalassemic patients and in healthy donors in Iraq: molecular and physiological study. PLoS Negl Trop Dis 14(6). 10.1371/journal.pntd.0007880 10.1371/journal.pntd.0007880 PMC7302744 32511233

[CR1] Beg MS, Brenner AJ, Sachdev J, Borad M, Kang YK, Stoudemire J, Hong DS (2017) Phase I study of MRX34, a liposomal miR-34a mimic, administered twice weekly in patients with advanced solid tumors. Invest New Drugs 35(2):180–188. 10.1007/s10637-016-0407-y 27917453 10.1007/s10637-016-0407-y PMC5893501

[CR2] Burgess JL, Wyant WA, Abujamra A, Kirsner B, R. S., Jozic I (2021) Diabetic Wound-Healing Science. Med (Kaunas) 57(10). 10.3390/medicina57101072 10.3390/medicina57101072 PMC8539411 34684109

[CR3] Cakmak H, Gokmen E, Bozkurt G, Kocaturk T, Ergin K (2018) Effects of sunitinib and bevacizumab on VEGF and miRNA levels on corneal neovascularization. Cutan Ocul Toxicol 37(2):191–195. 10.1080/15569527.2017.1375943 28874077 10.1080/15569527.2017.1375943

[CR4] Cerny MK, Wiesmeier A, Hopfner U, Topka C, Zhang W, Machens HG, Duscher D (2021) Wound fluid under occlusive dressings from diabetic patients show an increased angiogenic response and fibroblast migration. J Tissue Viability. 10.1016/j.jtv.2021.02.013 33707159 10.1016/j.jtv.2021.02.013

[CR6] Chen L, Gajendrareddy PK, DiPietro LA (2012) Differential expression of HIF-1α in skin and mucosal wounds. J Dent Res 91(9):871–876. 10.1177/0022034512454435 22821237 10.1177/0022034512454435 PMC3420394

[CR5] Chen K, Yu T, Wang X (2021a) Inhibition of circulating Exosomal miRNA-20b-5p accelerates Diabetic Wound Repair. Int J Nanomed 16:371–381. 10.2147/IJN.S287875 10.2147/IJN.S287875 PMC7813471 33469291

[CR7] Chen LH, Ma WX, Chen DW, Wang C, Gao Y, Ran XW (2021b) Association of high-density lipoprotein cholesterol and wound healing in patients with diabetic foot ulcers. Chin Med J (Engl). 10.1097/CM9.0000000000001544 33950872 10.1097/CM9.0000000000001544 PMC8850818

[CR8] Dallas A, Trotsyuk A, Ilves H, Bonham CA, Rodrigues M, Engel K, Johnston BH (2019) Acceleration of Diabetic Wound Healing with PHD2- and mir-210-Targeting oligonucleotides. Tissue Eng Part A 25(1–2):44–54. 10.1089/ten.TEA.2017.0484 29644938 10.1089/ten.tea.2017.0484 PMC6352500

[CR9] de Mattos RC, Guimaraes IDS, Thiago LS, de Melo AC (2021) Evaluation of HIF-1alpha and VEGF-A expression in radiation-induced cystitis: a case-control study. Int Braz J Urol 47(2):295–305. 10.1590/S1677-5538.IBJU.2020.0054 33146980 10.1590/S1677-5538.IBJU.2020.0054 PMC7857752

[CR10] Delfan M, Amadeh Juybari R, Gorgani-Firuzjaee S, Høiriis Nielsen J, Delfan N, Laher I, Zouhal H (2022) High-intensity interval training improves cardiac function by miR-206 dependent HSP60 induction in Diabetic rats. Front Cardiovasc Med 9:927956. 10.3389/fcvm.2022.927956 35845054 10.3389/fcvm.2022.927956 PMC9277013

[CR11] Deng L, Du C, Song P, Chen T, Rui S, Armstrong DG, Deng W (2021) The role of oxidative stress and antioxidants in Diabetic Wound Healing. Oxid Med Cell Longev 2021:8852759. 10.1155/2021/8852759 33628388 10.1155/2021/8852759 PMC7884160

[CR12] Falanga V (2004) The chronic wound: impaired healing and solutions in the context of wound bed preparation. Blood Cells Mol Dis 32(1):88–94. 10.1016/j.bcmd.2003.09.020 14757419 10.1016/j.bcmd.2003.09.020

[CR13] Gao Y, Yue J, Huang Z (2021) LncRNA MIAT mediates ox-LDL-Induced endothelial cell Injury Via miR-206/RAB22A Axis. J Surg Res 265:303–312. 10.1016/j.jss.2021.02.029 33965771 10.1016/j.jss.2021.02.029

[CR14] Geraghty T, LaPorta G (2019) Current health and economic burden of chronic diabetic osteomyelitis. Expert Rev Pharmacoecon Outcomes Res 19(3):279–286. 10.1080/14737167.2019.1567337 30625012 10.1080/14737167.2019.1567337

[CR15] Gouin JP, Kiecolt-Glaser JK (2011) The impact of psychological stress on wound healing: methods and mechanisms. Immunol Allergy Clin North Am 31(1):81–93. 10.1016/j.iac.2010.09.010 21094925 10.1016/j.iac.2010.09.010 PMC3052954

[CR16] Jodheea-Jutton A, Hindocha S, Bhaw-Luximon A (2022) Health economics of diabetic foot ulcer and recent trends to accelerate treatment. Foot (Edinb) 52:101909. 10.1016/j.foot.2022.101909 36049265 10.1016/j.foot.2022.101909

[CR17] Lin CJ, Lan YM, Ou MQ, Ji LQ, Lin SD (2019) Expression of miR-217 and HIF-1alpha/VEGF pathway in patients with diabetic foot ulcer and its effect on angiogenesis of diabetic foot ulcer rats. J Endocrinol Invest 42(11):1307–1317. 10.1007/s40618-019-01053-2 31079353 10.1007/s40618-019-01053-2

[CR18] Lin RX, Zhan GF, Wu JC, Fang H, Yang SL (2021) LncRNA SNHG14 sponges miR-206 to affect proliferation, apoptosis, and Metastasis of Hepatocellular Carcinoma Cells by regulating SOX9. Dig Dis Sci. 10.1007/s10620-021-06920-8 33782806 10.1007/s10620-021-06920-8

[CR19] Lu Q, Wang J, Wei X, Wang G, Xu Y, Lu Z, Liu P (2020) Cost of Diabetic Foot Ulcer Management in China: a 7-Year single-Center Retrospective Review. Diabetes Metab Syndr Obes 13:4249–4260. 10.2147/DMSO.S275814 33204131 10.2147/DMSO.S275814 PMC7667006

[CR20] Nie X, Zhao J, Ling H, Deng Y, Li X, He Y (2020) Exploring microRNAs in diabetic chronic cutaneous ulcers: Regulatory mechanisms and therapeutic potential. Br J Pharmacol 177(18):4077–4095. 10.1111/bph.15139 32449793 10.1111/bph.15139 PMC7443474

[CR21] Pan JY, Sun CC, Bi ZY, Chen ZL, Li SJ, Li QQ, Li DJ (2017) miR-206/133b cluster: A Weapon against Lung Cancer? Mol Ther Nucleic Acids 8:442–449. 10.1016/j.omtn.2017.06.002 28918043 10.1016/j.omtn.2017.06.002 PMC5542379

[CR22] Patel D, Chaudhary J (2012) Increased expression of bHLH transcription factor E2A (TCF3) in prostate cancer promotes proliferation and confers resistance to doxorubicin induced apoptosis. Biochem Biophys Res Commun 422(1):146–151. 10.1016/j.bbrc.2012.04.126 22564737 10.1016/j.bbrc.2012.04.126 PMC3361642

[CR23] Reid G, Kao SC, Pavlakis N, Brahmbhatt H, MacDiarmid J, Clarke S, van Zandwijk N (2016) Clinical development of TargomiRs, a miRNA mimic-based treatment for patients with recurrent thoracic cancer. Epigenomics 8(8):1079–1085. 10.2217/epi-2016-0035 27185582 10.2217/epi-2016-0035

[CR24] Rupaimoole R, Slack FJ (2017) MicroRNA therapeutics: towards a new era for the management of cancer and other diseases. Nat Rev Drug Discov 16(3):203–222. 10.1038/nrd.2016.246 28209991 10.1038/nrd.2016.246

[CR25] Saeedi P, Petersohn I, Salpea P, Malanda B, Karuranga S, Unwin N, Committee IDFDA (2019) Global and regional diabetes prevalence estimates for 2019 and projections for 2030 and 2045: results from the International Diabetes Federation Diabetes Atlas, 9(th) edition. Diabetes Res Clin Pract 157:107843. 10.1016/j.diabres.2019.107843 31518657 10.1016/j.diabres.2019.107843

[CR26] Spampinato SF, Caruso GI, De Pasquale R, Sortino MA, Merlo S (2020) The treatment of impaired Wound Healing in Diabetes: looking among old drugs. Pharmaceuticals (Basel) 13(4). 10.3390/ph13040060 10.3390/ph13040060 PMC7243111 32244718

[CR27] Vinod M, Patankar JV, Sachdev V, Frank S, Graier WF, Kratky D, Kostner GM (2016) MiR-206 is expressed in pancreatic islets and regulates glucokinase activity. Am J Physiol Endocrinol Metab 311(1):E175–E185. 10.1152/ajpendo.00510.2015 27221121 10.1152/ajpendo.00510.2015 PMC4941929

[CR28] Wang W, Yang C, Wang XY, Zhou LY, Lao GJ, Liu D, Ren M (2018) MicroRNA-129 and – 335 promote Diabetic Wound Healing by inhibiting Sp1-Mediated MMP-9 expression. Diabetes 67(8):1627–1638. 10.2337/db17-1238 29748291 10.2337/db17-1238

[CR29] Xiong Y, Chen L, Yan C, Zhou W, Endo Y, Liu J, Liu G (2020) Circulating Exosomal miR-20b-5p inhibition restores Wnt9b Signaling and Reverses Diabetes-Associated impaired Wound Healing. Small 16(3):e1904044. 10.1002/smll.201904044 31867895 10.1002/smll.201904044

[CR30] Xu Z, Zhu C, Chen C, Zong Y, Feng H, Liu D, Lu A (2018) CCL19 suppresses angiogenesis through promoting miR-206 and inhibiting Met/ERK/Elk-1/HIF-1alpha/VEGF-A pathway in colorectal cancer. Cell Death Dis 9(10):974. 10.1038/s41419-018-1010-2 30250188 10.1038/s41419-018-1010-2 PMC6155262

[CR31] Xue D, Yang Y, Liu Y, Wang P, Dai Y, Liu Q, Tan Z (2016) MicroRNA-206 attenuates the growth and angiogenesis in non-small cell lung cancer cells by blocking the 14-3-3zeta/STAT3/HIF-1alpha/VEGF signaling. Oncotarget 7(48):79805–79813. 10.18632/oncotarget.12972 27806334 10.18632/oncotarget.12972 PMC5346752

[CR32] Yamaura Y, Kanki M, Sasaki D, Nakajima M, Unami A (2020) Serum miR-206 as a biomarker for drug-induced skeletal muscle injury in rats. J Toxicol Sci 45(8):503–513. 10.2131/jts.45.503 32741900 10.2131/jts.45.503

[CR34] Yang S, Evens AM, Prachand S, Singh AT, Bhalla S, David K, Gordon LI (2010) Mitochondrial-mediated apoptosis in lymphoma cells by the diterpenoid lactone andrographolide, the active component of Andrographis paniculata. Clin Cancer Res 16(19):4755–4768. 10.1158/1078-0432.CCR-10-0883 20798229 10.1158/1078-0432.CCR-10-0883 PMC2948634

[CR35] Yang ZG, Awan FM, Du WW, Zeng Y, Lyu J, Wu,. Yang BB (2017) The circular RNA interacts with STAT3, increasing its Nuclear translocation and wound repair by modulating Dnmt3a and miR-17 function. Mol Ther 25(9):2062–2074. 10.1016/j.ymthe.2017.05.022 28676341 10.1016/j.ymthe.2017.05.022 PMC5589065

[CR33] Yang L, Liu N, Zhao W, Li X, Han L, Zhang Z, Mao B (2019) Angiogenic function of astragaloside IV in rats with myocardial infarction occurs via the PKD1-HDAC5-VEGF pathway. Exp Ther Med 17(4):2511–2518. 10.3892/etm.2019.7273 30906439 10.3892/etm.2019.7273 PMC6425153

[CR36] Yu L, Li J, Peng B, Cai P, Zhao B, Chen Y, Zhu H (2021) CircASXL1 Knockdown Restrains Hypoxia-Induced DDP Resistance and NSCLC Progression by sponging miR-206. Cancer Manag Res 13:5077–5089. 10.2147/CMAR.S276964 34234552 10.2147/CMAR.S276964 PMC8253994

[CR37] Zhu Y, Wang Y, Jia Y, Xu J, Chai Y (2019) Roxadustat promotes angiogenesis through HIF-1alpha/VEGF/VEGFR2 signaling and accelerates cutaneous wound healing in diabetic rats. Wound Repair Regen 27(4):324–334. 10.1111/wrr.12708 30817065 10.1111/wrr.12708

